# Fructans from *Agave tequilana* with a Lower Degree of Polymerization Prevent Weight Gain, Hyperglycemia and Liver Steatosis in High-Fat Diet-Induced Obese Mice

**DOI:** 10.1007/s11130-016-0578-x

**Published:** 2016-09-27

**Authors:** A. L. Márquez-Aguirre, R. M. Camacho-Ruíz, Y. K. Gutiérrez-Mercado, E. Padilla-Camberos, M. González-Ávila, F. J. Gálvez-Gastélum, N. E. Díaz-Martínez, D. Ortuño-Sahagún

**Affiliations:** 1 Unidad de Biotecnología Médica y Farmacéutica, Centro de Investigación y Asistencia en Tecnología y Diseño del Estado de Jalisco, A.C., Guadalajara, Jalisco Mexico; 2 Unidad de Biotecnología Industrial, Centro de Investigación y Asistencia en Tecnología y Diseño del Estado de Jalisco, A.C., Guadalajara, Jalisco Mexico; 3 Laboratorio de Patología, CUCS, Universidad de Guadalajara, Guadalajara, Jalisco Mexico; 4 Instituto de Investigación en Ciencias Biomédicas (IICB), CUCS, Universidad de Guadalajara, Guadalajara, Jalisco Mexico

**Keywords:** Dietary fiber, Fructans, Agave, Liver steatosis

## Abstract

Fructans from agave have received specific attention because of their highly branched fructan content. We have previously reported that the degree of polymerization (dp) influences their biological activity. Therefore, the aim of this study was to investigate the effect of unfractionated and fractionated fructans (higher and lower dps) from *Agave tequilana* in high-fat diet-induced (HFD) obese mice. Fructans with a lower dp (HFD+ScF) decreased weight gain by 30 %, body fat mass by 51 %, hyperglycemia by 25 % and liver steatosis by 40 %. Interestingly, unfractionated fructans (HFD+F) decreased glucose and triglycerides (TG), whereas fractionated fructans with a higher dp (HFD+LcF) decreased TG but not glucose; in contrast, HFD+ScF decreased glucose but not TG. Our findings suggest that both higher and lower dp agave fructans have complementary effects in metabolic disorders related to obesity. These findings may contribute to the development of improved food supplements with a specific ratio combination of fructans with different dps.

**Electronic supplementary material:**

The online version of this article (doi:10.1007/s11130-016-0578-x) contains supplementary material, which is available to authorized users.

## Introduction

Dietary fibers, such as inulin-type fructans, are selectively used and fermented by the gut, and several studies have demonstrated their health benefits [1–4]. For example, these non-digestible carbohydrates have been demonstrated to reduce weight gain and related metabolic disorders *via* specific actions on food intake [5–7]. Most of these findings have been obtained following supplementation with inulin-type fructans from Jerusalem artichoke (*Helianthus tuberosus*) and chicory (*Cichorium intybus*) [8]. The inulin from these sources comprises linear chains of β (2–1) fructans (unbranched). Interestingly, *Agave tequilana* Weber var. azul contains complex fructans with demonstrated bioactivity. These fructans are highly branched with both beta (2–1) and beta (2–6) linkages [9, 10]. Moreover, they are resistant to hydrolysis by human digestive enzymes and may be fermented by colonic microbiota; however, their mode of action is not completely elucidated. Fructans from *Agave tequilana* and *Agave angustifolia* have been primarily investigated as a complex mix of different chain lengths (total fructans). C57Bl/6J mice fed a standard diet supplemented with fructans from *Agave tequilana* Gto., exhibited reduced food intake, body weight and plasma glucose and lipids [11]. In diabetic rats with normal weight, fructans from *Agave angustifolia* diminished hyperglycemia and liver steatosis [12]. A previous study conducted in our laboratory demonstrated that both the degree of polymerization (dp) and the demineralization process influence the biological activity of agave fructans. Our studies indicate that the treatment of obese mice with fructans with a lower dp (dp < 10) from *A. tequilana* did not increase the count of fecal *Bifidobacteria*; however, it reduced body weight. In contrast, obese mice that received total fructans exhibited an increased fecal *Bifidobacteria* count; however, they did not exhibit changes in the lipid profile or body weight [13]. It was subsequently reported that agavins (agave fructans) with a lower dp from *A. angustifolia* and *A. potatorum* promoted the release of peptides involved in appetite regulation and may thus be involved in the control of obesity and its associated metabolic disorders [14]. On this basis, the present study aimed to investigate the effect of unfractionated and fractionated fructans (higher and lower dp) from *Agave tequilana* in high-fat diet-induced obese mice.

## Materials and Methods

### Agave Fructan Extraction, Purification and Carbohydrate Profile Characterization

*Agave tequilana* Weber var. azul plants were collected from an endemic growing area in Tequila, Jalisco, Mex. The collection, purification and characterization were performed as previously described [13]. The total contents of the fructans were labeled F, whereas two fractions of fructans with different dp profiles were obtained *via* ultrafiltration procedures. Agave fructans with a higher dp >10 (labeled LcF) were obtained from the retentate of a 3 kDa membrane, whereas fructans with a lower dp < 10 (labeled ScF) were recovered from the retentate of a 1 kDa membrane.

### Animals, Diets and Experimental Groups

Sixty male mice (C57/BL/6) with body weights of 20–25 g (Harlan/Envigo Inc.) were housed in a controlled environment with free access to food and water. After one week of acclimatization, the mice were randomly divided into two experimentation groups. A) Model validation; with two groups (*n* = 10/group): 1) was fed a standard diet (SD) with 18 % fat (energy density 3.1 kcal/g), and 2) was fed a high fat diet (HFD) with 60 % fat (energy density 5.1 kcal/g), both for eight weeks (20128S and TD.6414 diets, respectively, from Teklad Harlan/Envigo Inc.). B) Treatments; with four groups (*n* = 10/group): 3) Orafti sinergy1^TM^ (HFD+OS1), unbranched inulin-type fructans from chicory, 4) unfractionated agave fructans (HFD+F) and fractionated, 5) agave fructans with a higher dp (HFD+LcF) and 6) a lower dp (HFD+ScF). The mice had free access to the HFD or SD in common cages (five per cage); however, they were orally administered with fructans at doses of 125 mg / 25 g of body weight individually three times *per* week for eight weeks. The study was approved by the local Animal Care and Use Committee and complied with local (NOM-062-ZOO-1999) and International Guidelines (Animal Welfare Assurance A5281-01).

### Biochemical Assays

The fasting glucose concentration was determined from tail vein blood using a hand-held glucometer, ONETOUCH® ULTRA®, with reactive strips (Johnson & Johnson). At the end of the experiment, the total cholesterol (TC), triglycerides (TG), high-density lipoproteins (HDL), low-density lipoproteins (LDL), very low-density lipoproteins (VLDL) and hepatic enzymes AST and ALT were measured *via* automated enzymatic methods on a Sincron-7 analyzer.

### Oral Glucose Tolerance Test

An oral glucose tolerance test was performed on 6 h fasting-mice [15]. In brief, the mice were administered glucose *via* gavage (1 g/kg glucose, 20 % glucose solution). The blood glucose was determined using reactive strips on a glucose meter from whole blood drawn from the tail-tip capillary region at points 0, 30, 60 and 120 min after gavage.

### Fat Pad Collection and Liver Histology

The mice were sacrificed *via* an i.p. injection with ketamine / xylazine (100/10 mg / kg). White adipose tissues were dissected and weighed. Liver tissues were stained with hematoxylin and eosin and observed under a light microscope (Leica, DMR). Biopsies were classified into three grades [16], in which a sample is classified as grade 1 when fat vacuoles are identified in 5–33 % of hepatocytes, grade 2 when 33–66 % of hepatocytes are affected by fat vacuoles, and grade 3 when fat vacuoles are identified in >66 % of hepatocytes.

### Data Analyses

A Kolmogorov-Smirnov test was performed for all data to determine whether the values originate from a Gaussian distribution. In the first experiment “Model validation”, statistical differences between groups were assessed using unpaired *t*-tests two-tailed, whereas for the second experiment “Treatments”, statistical differences between groups were assessed using one-way ANOVA followed by a Dunnett *post-hoc* test to compare all treatments *vs.* HDF with GraphPad Prism 5 Software, Inc., USA. Differences were considered significant at *p* ≤ 0.05. The obtained data are expressed as the mean ± standard deviation (mean ± SD).

## Results and Discussion

**Fig. 1:**
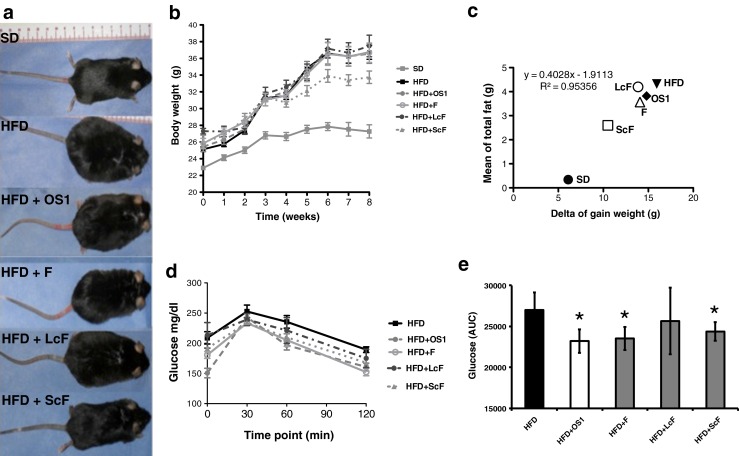
Growth performance and glycemia. Final weight (**a**), body weight kinetics (**b**), correlation of gain weight and body fat (**c**), glucose tolerance test and AUC (**d** and **e**) in mice fed a HFD and supplemented with fructans: chicory fructans (OS1), agave fructans unfractionated (F) and fractionated in a higher dp (LcF) and lower dp (ScF). Data are presented as the mean ± SD (*n* = 10). The *asterisk* (*) denotes a significant difference compared with a HFD (*p* < 0.05)

A limited number of studies have utilized branched fructans from agave. These studies were primarily designed to analyze the prebiotic effects from agave fructans in *in vitro* studies [17–21], animal models [13, 22, 23] and clinical trials [24–27], whereas other studies have investigated the effects on food intake, body weight gain and hyperglycemia [11, 14, 22, 28]. Only one study has been conducted to analyze liver steatosis in diabetic rats supplemented with agave fructans [12], and several studies have been designed to investigate the relationship between dp and biological activity [13, 20, 21]. Thus, our study aimed to investigate the effect of unfractionated and fractioned branched fructans (higher and lower dp) from *Agave tequilana* in HFD induced obese mice and compare them with unbranched –linear- chicory fructans. As expected, the final body weight, weight gain and fat mass in the mice fed a HFD were increased (*p* < 0.05) compared with the mice fed a SD (supplementary file 1). The weight gain in the HFD was approximately 10 g more than the SD, whereas the fat mass was increased more than 10-fold. The obese mice accumulated more epididymal (1.86 g) and subcutaneous (1.78 g) fat compared with visceral fat (0.68 g). The mice fed a SD attained a total fat accumulation of 0.34 g, whereas the mice fed a HFD accumulated 4.32 g (11.7-fold, compared with the mice fed a SD). The blood glucose, TG, TC and AST concentrations of the HFD mice were also increased (*p* < 0.05) compared with the SD mice. Finally, the mice fed a HFD exhibited substantially increased blood glucose, 60 mg/dl more than the SD, whereas the TG and TC increases were 100 and 80 mg / dl, respectively. Commercially inulin-type fructans from chicory (unbranched fructans) were used as a reference because they have been extensively investigated as prebiotics and may be used to compare branched fructans from agave. However, we did not identify statistically significant differences in the weight gain, fat mass, lipid profile or transaminases in the obese mice treated with unbranched fructans (HFD+OS1) (Table 1). Fructans from *Agave tequilana* with a lower dp (HFD+ScF) decreased (*p* ≤ 0.05) the final body weight (Table 1, Fig. 1a and b), weight gain and fat mass (Table 1). These animals exhibited a weight difference of 7.4 g compared with the HFD, which represents a decrease of 30 % in the body weight gain. HFD+ScF also accumulated 51 % less fat compared with the mice fed a HFD, which resulted in 2.28 g of total fat. We identified a positive linear correlation between the weight increase and fat pad accumulation in all treatments (Fig. 1c) with an R2 of 0.9535; however, the HFD+ScF showed reduced (*p* ≤ 0.05) epididymal and subcutaneous fat even if they were simultaneously fed a HFD. These findings indicate that ScF from *Agave tequilana* significantly reduces body weight gain by diminishing fat accumulation in obese mice. In contrast, unfractionated agave fructans (HFD+F) and agave fructans with a higher dp (HFD+LcF) did not exhibit statistical differences in body weight, weight gain or fat mass; however, TG decreased (*p* ≤ 0.05). Fructan supplementation with inulin-type fructans from chicory (HDF+OS1), unfractionated fructans (HFD+F) and fractionated fructans with a lower dp from agave (HFD+ScF) decreased (*p* ≤ 0.05) glucose and the area under the curve (AUC) in the oral glucose tolerance test (between 14 and 45 %) compared with the AUC of the obese mice fed a HFD. The obese mice exhibited a concentration of 171 mg / dl, whereas treatment with fructans from chicory (HFD+OS1) decreased hyperglycemia by 19 % (139 mg / dl), the treatment with agave unfractionated fructans (HFD+F) decreased hyperglycemia by 20 % (137 mg/dl) and fractionated fructans with lower dp (HFD+ScF) decreased hyperglycemia by 25 % (129 mg / dl) and the AUC was 24,375 units compared with 27,000 units in the obese mice fed a HFD (Table 1, Fig. 1d and e). In summary, unbranched fructans reduced glycemia; however, there was no change in the serum cholesterol and TG levels. Similar results have been demonstrated in dietary supplementation with inulin in rats fed a HFD [7]. Moreover, the mice fed branched fructans from *A. tequilana* with a lower dp (HFD+ScF) decreased body weight gain by 30 %, body fat mass by 51 % and glycemia by 25 %. Interestingly, unfractionated branched fructans (HFD+F) decreased blood glucose and TG, whereas fractionated fructans with a higher dp (HFD+LcF) decreased TG but not glucose; moreover, fructans with a lower dp (HFD+ScF) decreased blood glucose but not TG. Linear fructans from chicory exhibited similar effects on glucose to unfractionated branched fructans from agave. These findings may explain the evidence regarding reductions in weight gain and blood glucose concentration in different animal models supplemented with total fructans from *Agave* spp., including non-obese mice [11, 14], diabetic rats [12] and a hypercholesterolemic model [28]. C57BL/6J male mice fed a HFD share nearly the same human obesity phenotype; visceral adiposity, hyperglycemia, insulin and leptin resistance, as well as hepatic steatosis [29, 30]. In this study, the liver steatosis grades were affected by fructan supplementation (supplementary file 2). Treatment with unbranched fructans from chicory (HFD+OS1) reduced accumulated fat droplets (macrovesicular), which decreased the steatosis percentage (20 %) similar to the animals supplemented with fructans from agave with a higher dp (HFD+LcF). Moreover, treatment with unfractionated fructans (HFD+F) exhibited a less decrease in the degree of steatosis (10 %). The most interesting results were identified in the mice treated with fructans from agave with a lower dp (HFD+ScF), which indicated reduced steatosis (microvesicular) (40 %) with minimal inflammatory invasions and few hypertrophic hepatocytes (grade 1) compared with the untreated mice (HFD) with moderate steatosis (grade 2). These findings are consistent with the reduction in AST levels presented in Table 1 and could explain why liver steatosis decreased in diabetic rats supplemented with fructans from *Agave angustifolia* as previously reported [12]. Bacteria grew according to the molecular weight of the agave fructans [20], in which fructans with a higher dp exert a more pronounced prebiotic effect [31] in contrast to fructans with a lower dp that exhibit more anti-obesity effects [12, 13].

**Table 1:** Effects of fructans on weight gain, body fat, lipid profile and liver enzymes in mice fed a HFD for eight weeks

	Obese	Treatments			
		Linear chicory fructans	Branched Agave fructans		
			Unfractionated	Fractionated	
	HFD	HFD + OS1	HFD + F	HFD + LcF	HFD + ScF
Final body weight (g)	36.71 ± 1.5	36.55 ± 3.6	35.52 ± 3.7	37.49 ± 4.2	33.5 ± 2.2*
Gain weight (g)	16.37 ± 2.0	13.26 ± 1.0	12.54 ± 0.82	11.92 ± 1.2	8.96 ± 0.42*
Fat Mass (g)	4.32 ± 0.25	3.82 ± 0.62	4.48 ± 0.37	4.20 ± 0.42	2.28 ± 0.52*
Epididymal (g)	1.86 ± 0.10	1.68 ± 0.30	1.85 ± 0.14	2.04 ± 0.21	1.04 ± 0.15*
Visceral (g)	0.67 ± 0.03	0.62 ± 0.09	0.51 ± 0.05	0.65 ± 0.06	0.42 ± 0.11
Subcutaneous (g)	1.78 ± 0.18	1.50 ± 0.25	1.19 ± 0.11	1.50 ± 0.15	0.71 ± 0.19*
Triglycerides (mg/dl)	196.0 ± 60.4	136.6 ± 8.8	95.0 ± 5.5*	92.6 ± 5.2*	129.6 ± 9.8
Total cholesterol (mg/dl)	147.6 ± 8.5	140.0 ± 13.0	151.3 ± 2.1	145.6 ± 5.2	124.3 ± 3.1
HDL (mg/dl)	71.3 ± 10.9	80.0 ± 5.0	79.3 ± 4.0	70.6 ± 8.7	55.3 ± 8.5
LDL (mg/dl)	37.0 ± 10.6	32.67 ± 10.4	53.0 ± 2.6	56.3 ± 14.1	43.3 ± 9.3
VLDL (mg/dl)	39.3 ± 11.9	27.3 ± 1.6	19.0 ± 1.1	18.6 ± 0.8	25.6 ± 2.0
Glucose (mg/dl)	171.0 ± 17.1	139.1 ± 19.5*	137.1 ± 14.11*	160.4 ± 20.5	129.1 ± 22.4*
AST (U/l)	159.0 ± 9.6	134.6 ± 22.5	146.6 ± 28.3	101.6 ± 21.2	66.3 ± 8.5*
ALT (U/l)	64.6 ± 9.6	51.3 ± 11.6	46.3 ± 4.4	52.3 ± 1.4	47.0 ± 2.3

Mean ± SD, growth parameters *n* = 10, biochemical parameters *n* = 6. * *p* < 0.05

*Abbreviations*: *ALT* alanine aminotransferase, *AST* aspartate aminotransferase, *F* total fructans, *HFD* high-fat diet, *HDL* high-density lipoproteins, *LcF* fructans with higher dp, *LDL* low-density lipoproteins, *OS1* Orafti sinergy1, *ScF* fructans with lower dp, *SD* standard-diet, *VLDL* very low-density lipoproteins

## Conclusions

Our findings suggest that branched fructans from agave with a lower dp are responsible for most of the beneficial effects exerted by unfractionated fructans and represent a powerful tool to prevent body weight gain, fat accumulation, liver steatosis and hyperglycemia, despite high fat diet consumption. However, both higher and lower dp agave fructans have complementary effects in metabolic disorders related to obesity. A high concentration of lower dp fructans is required to achieve a reduction in weight gain and liver steatosis. Therefore, it would be desirable to have agave branched fructans in a specific ratio of higher and lower dp to achieve positive effects in all metabolic disorders related to obesity, and studies on this effective ratio should be performed.

## Electronic supplementary material

Below is the link to the electronic supplementary material.

Supplementary file 1 (PDF 22 kb)

Supplementary file 2 (PDF 8420 kb)

## Abbreviations

ALT: Alanine aminotransferase
AST: Aspartate aminotransferase
dp: Degree of polymerization
F: Total fructans
HFD: High-fat diet
LcF: Fructans with a higher dp
OS1: Orafti sinergy1
ScF: Fructans with a lower dp
SD: Standard-diet
TG: Triglycerides
TC: Total cholesterol
