# Supplementary material for: Fructans from Agave tequilana with a Lower Degree of Polymerization Prevent Weight Gain, Hyperglycemia and Liver Steatosis in High-Fat Diet-Induced Obese Mice
Source: Plant Foods Hum Nutr. 2016 Sep 27;71(4):416–21. doi: 10.1007/s11130-016-0578-x (PMC5116042; doi:10.1007/s11130-016-0578-x)
Supplement: Supplementary file 1 — (PDF 22 kb) [file 11130_2016_578_MOESM1_ESM.pdf]

Supplementary file 1: Table. Body weight, body fat, lipid profile, liver enzymes and glucose in C57BL/6 mice fed a SD or HFD for 8 weeks

	**Non-obese**	**Obese**	
	**Standard Diet (SD)**	**High-Fat Diet (HFD)**	**p value**
Final body weight (g)	27 ± 0.6	36.7 ± 0.8	0.006
Gain weight (g)	6.16 ± 0.7	15.9 ± 0.8	0.0001
Fat Mass (g)	0.34 ± 0.03	4.32 ± 0.2	0.0001
Epididymal (g)	0.2 ± 0.02	1.86 ± 0.1	0.001
Visceral (g)	0.08 ± 0.02	0.68 ± 0.03	0.001
Subcutaneous (g)	0.06 ± 0.01	1.78 ± 0.2	0.003
Triglycerides (mg/dl)	94 ± 6.6	196 ± 60.4	0.01
Total cholesterol (mg/dl)	82 ± 14.5	147.7 ± 8.5	0.01
HDL (mg/dl)	54 ± 4.0	71.3 ± 10.9	NS
LDL (mg/dl)	18.7 ± 3.7	37 ± 10.7	NS
VLDL (mg/dl)	18.7 ± 1.3	39.3 ± 11.9	NS
Glucose (mg/dl)	110.6 ± 8.1	171 ± 5.9	0.0002
AST (U/l)	64.7 ± 6.9	159 ± 9.6	0.01
ALT (U/l)	50.3 ± 2.8	64.7 ± 9.7	NS

Mean ± SD, in growth parameters n=10 and biochemical parameters n=6. Abbreviations: ALT, alanine aminotransferase; AST, aspartate aminotransferase; HFD, High-Fat Diet; HDL, high-density lipoproteins; LDL, low-density lipoproteins; SD, standard-Diet; VLDL, Very low-density lipoproteins
