# Supplementary material for: Fructans from Agave tequilana with a Lower Degree of Polymerization Prevent Weight Gain, Hyperglycemia and Liver Steatosis in High-Fat Diet-Induced Obese Mice
Source: Plant Foods Hum Nutr. 2016 Sep 27;71(4):416–21. doi: 10.1007/s11130-016-0578-x (PMC5116042; doi:10.1007/s11130-016-0578-x)
Supplement: Supplementary file 2 — (PDF 8420 kb) [file 11130_2016_578_MOESM2_ESM.pdf]

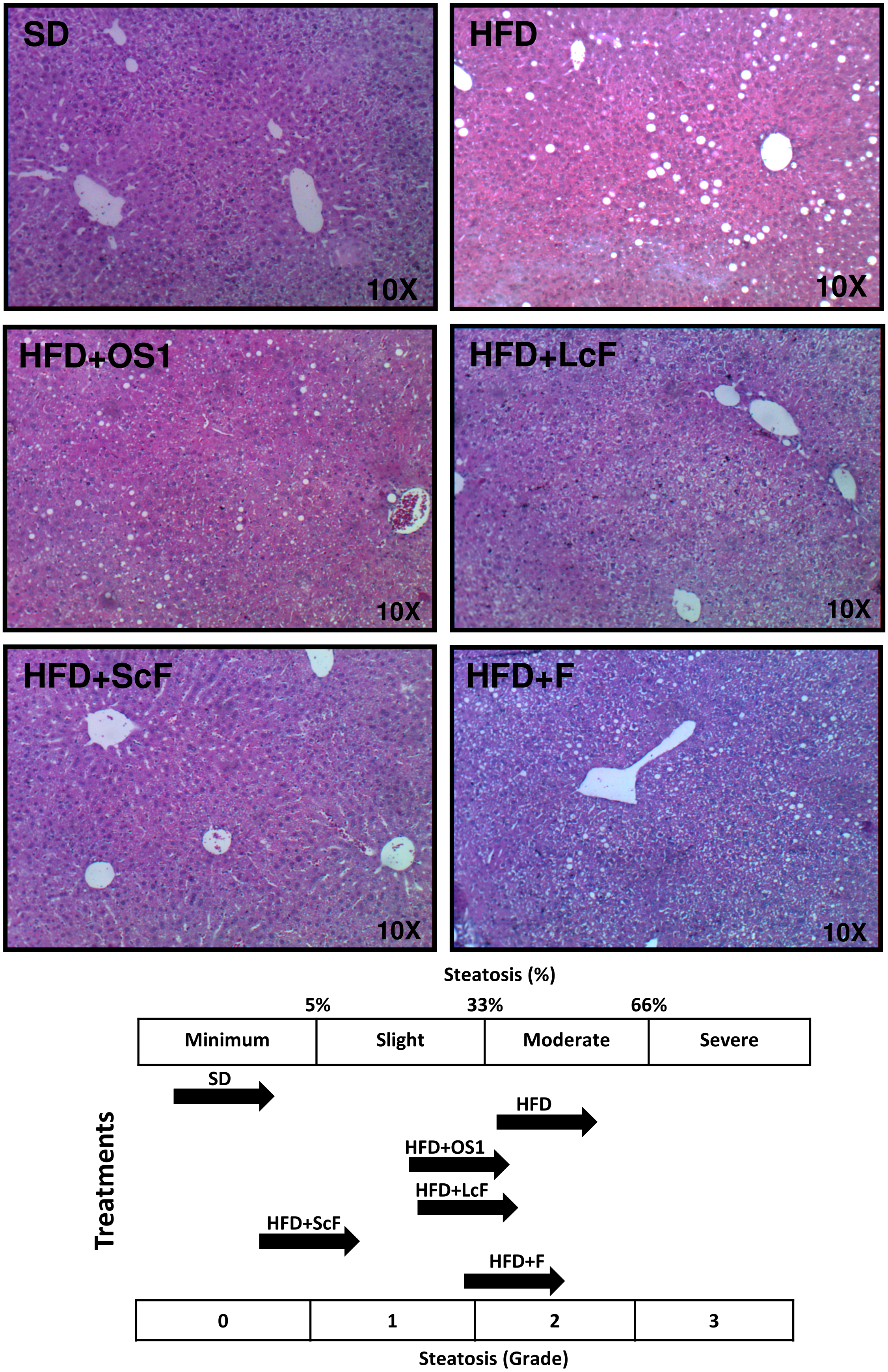

Supplementary file 2: Figure. Liver steatosis. Representative photomicrographs of liver stained with hematoxylin-eosin. Mice fed a HFD and supplemented with fructans: chicory fructans (OS1), agave fructans unfractionated (F) and fractionated in a higher dp (LcF) and lower dp (ScF). Microvesicular vacuolation (steatosis) of hepatocyte cytoplasm characterized by clear, round vacuoles.
